# Investigating interindividual variations in cortical bone quality: analysis of the morphotypes of secondary osteons and their population densities in the human femoral diaphysis

**DOI:** 10.1007/s12565-018-0452-z

**Published:** 2018-07-30

**Authors:** Hiroaki Matsuo, Toshiyuki Tsurumoto, Junichiro Maeda, Kazunobu Saiki, Keishi Okamoto, Keiko Ogami-Takamura, Hisayoshi Kondo, Masato Tomita, Akihiko Yonekura, Makoto Osaki

**Affiliations:** 1 Department of Orthopaedic Surgery, Nagasaki University Graduate School of Biomedical Sciences, 1-12-4 Sakamoto, Nagasaki City, Nagasaki Prefecture 852-8523 Japan; 2 Department of Macroscopic Anatomy, Nagasaki University Graduate School of Biomedical Sciences, Nagasaki City, Japan; 3 Biostatistics Section, Division of Scientific Data Registry, Atomic Bomb Disease Institute, Nagasaki University, Nagasaki City, Japan

**Keywords:** Osteon, Femoral diaphysis, Cortical bone, Morphotype, Bone quality

## Abstract

Osteons are the primary sites of cortical bone lesions. However, many aspects of osteon microstructure remain poorly understood. This study aimed to explores interindividual differences in the osteon morphotype distributions in the human femoral diaphysis by evaluating the secondary osteon distributions in samples from human femurs. Two anonymized bone fragments from two modern Japanese femurs were examined. Twelve continuous transverse femoral diaphysis specimens were prepared from each fragment. Imaging examinations were conducted using a circularly polarized light microscope, and cross-sectional images were rendered using graphical synthesis software. Osteons in the images were identified as either bright-type osteons, dark-type osteons, or an others type. The two femurs were compared, and the secondary osteon morphotype distributions in different regions of their cross-sections were analyzed. When the two femurs were compared, significant differences in osteon density were observed in some regions and cross-sections. The dark-type osteon presence was strongest in the anterior and posterior regions of the femurs. The analytical method used in this study was found to be able to evaluate osteon microstructure. The results suggest that examining additional specimens and analyzing the biomechanical underpinnings of interindividual differences in osteon distribution patterns may help to improve our understanding of osteon microstructure.

## Introduction

Osteons are the main sites of cortical bone remodeling. Osteons were formed either within or at the periosteum on existing bone. Primary osteons were generated during appositional bone growth, whereas secondary osteons are generated during internal bone remodeling. Since 1999, Bell et al. (1999) have focused their studies on the diameter of the central canals of osteons that constitute the femoral neck cortex, and they have found that fractures are likely to occur in regions where osteons with relatively large central canals are distributed, leading to the concept of “cortical porosity.” They have also introduced the concept of “cortical bone trabecularization,” based on the findings of a morphological analysis of the central canals of osteons. They observed that cortical porosity tended to occur in regions where the proportion of secondary osteons was high—regions undergoing bone remodeling.

Variations in the layering of the collagen fibers that constitute the lamellar structure of osteons have been observed using a circularly polarized light microscope. Gebhardt (1906) described images of the collagen fiber structure in osteons obtained using circularly polarized light microscopy, and Ascenzi and Bounucci (1967, 1968, 1972) later observed the microstructure of the human femur and proposed three different osteon morphotype (OM) classifications, including longitudinal-type (dark-type) osteons, in which the collagen fibers are aligned parallel to the longitudinal axis of the femoral diaphysis and are resistant to tension, and transversal type (bright-type) osteons, in which the collagen fibers are aligned in a spiral transverse to the axis of the femoral diaphysis and therefore resist compression forces. Numerous studies in recent years have examined osteon structure using circularly polarized light microscopy. Bromage et al. (2003) classified osteons into three morphotypes (bright, dark, and alternating) and Bigley et al. (2006) classified them into four groups, adding a hooped type. Beraudi et al. (2010) modified the OM classification system proposed by Bigley et al. (2006) and evaluated images of osteons from the diaphysis of the human fibula. Also, numerous studies have found that osteon size decreases with age, while the overall osteon population density (OPD) in cortical bone tissue increases with age (Britz et al. 2009; Currey 1964; Kerley 1965; Maat et al. 2006; Martin et al. 1980). Currey (1964) reported that the haversian system shrinks with age, and Martin et al. (1980) stated that osteons may decrease in size with age because of diminishing osteoclast activity. In addition, Britz et al. (2009) reported that age, sex, and body weight may also influence osteon size. The high level of bone remodeling observed in regions with elevated osteon density (Miszkiewicz 2016) may be associated with mineral homeostasis maintenance, bone microdamage repair, and adaptations to changes in the mechanical burden (Burr 2002). The femur is affected by load stress during both standing and walking, and the muscles attached to the femur continuously exert mechanical forces in various directions (Duda et al. 1997; Rybicki et al. 1972). However, muscle mass and resiliency slowly decline with age (Burr 1997; Frost 1997). These factors give rise to qualitative and quantitative changes in the femoral cortical bone. The aim of the study reported in the present paper was therefore to explore interindividual differences in the age-related fragility of femoral cortical bones by evaluating both the population density of secondary osteons and the structural patterns of secondary osteon morphotypes.

## Materials and methods

### Study samples

**Fig. 1:**
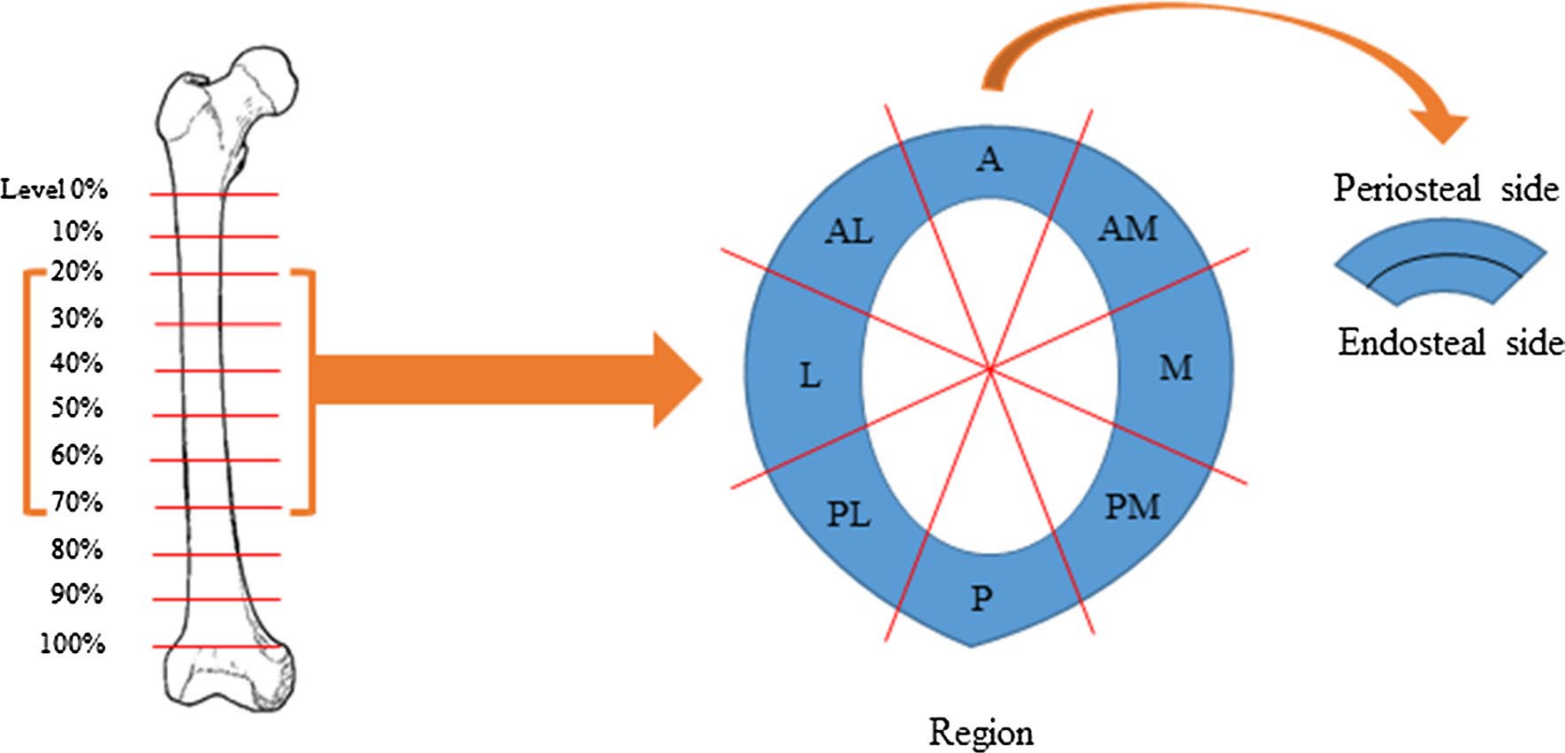
Designating the specimen cross-sectional levels, regions, and sides. A total of 11 cross-sectional levels equally spaced throughout the diaphyseal region (10, 20, 30, 40, 50, 60, 70, 80, and 90%) were designated. In this study, six of those cross-sectional levels (20, 30, 40, 50, 60, and 70%) were evaluated (*left*). Adopting the center of the medullary canal as a fixed point, eight equally divided regions of each cross-section were established: the anterior (*A*), anterior medial (*AM*), medial (*M*), posterior medial (*PM*), posterior (*P*), posterior lateral (*PL*), lateral (*L*), and anterior lateral (*AL*) regions. Furthermore, each of these eight regions had both a periosteal side and an endosteal side (*right*)

Twelve cross-sections from two randomly selected femurs from the skeletal specimens of modern Japanese citizens stored at the Nagasaki University of Medicine were evaluated in this study. These were obtained from cadavers used for anatomical dissection by medical students during the 1950s, 1960s, and 1970s. Most of the cadavers were donated voluntarily and were anonymous subjects. Subjects with obvious trauma or inflammatory joint disease were excluded from this study. None of the subjects would have taken drugs for osteoporosis before they died. Based on an assessment of their morphological characteristics, the donors of the two femurs were both male. By applying the age estimation method proposed by Kerley (1965) and Maat et al. (2006), the donor of femur A was estimated to have been 53 ± 5 years old when he died, and the donor of femur B was estimated to have been 33 ± 5 years old at death. In the diaphyses of these two femur specimens, the cross-section orthogonal to the bone axis through the base of the lesser trochanter was defined as the 0% level, and the cross-section orthogonal to the bone axis through the tip of the adductor muscle node in the upper region of the condyle was defined as the 100% level. In addition, a total of 11 cross-sectional levels equally spaced throughout the diaphyseal region (0, 10, 20, 30, 40, 50, 60, 70, 80, 90, and 100%) were designated. Both femurs were right-side femurs, and the lengths from the 0% to the 100% level of femurs A and B were measured as 280 mm and 294 mm, respectively. In this study, six cross-sectional levels (20, 30, 40, 50, 60, and 70%) were evaluated (Fig. 1, left). The mean cross-sectional area of femur A across six levels was 394.323 mm^2^, and that of femur B was 365.228 mm^2^.

### Specimen creation and circularly polarized light microscopy imaging

Each femur was divided at 10% intervals using a band saw (TBS-50, Ryobi, Hiroshima, Japan) to produce blocks that were approximately 5 mm in length. Each block was immersed in a photopolymerization-reactive resin (Technovit 7200 VLC^®^, Kulzer, LLC, South Bend, IN, USA) and irradiated with ultraviolet light for 3 days. The resin-impregnated bone specimen blocks were affixed to a polymethyl methacrylate (PMMA) plate using an instant glue (Aron Alpha 801^®^, Toagosei Co., Ltd., Tokyo, Japan), and were then manually polished to 500 μm using a whetstone (#100, #600). Next, abrasive specimens with a thickness of 100 μm were prepared using abrasive paper (#2000, #4000, #8000), and the polishing operation was continued while the thickness of the specimen was confirmed using a digital caliper (Mitutoyo Co. Ltd., Kawasaki, Japan). Following specimen polishing, images of the block cross-sections were taken at low magnification using a circularly polarized light microscope (BX 43, Olympus, Tokyo, Japan; Axiocam ERc 5s, Zeiss, Oberkochen, Germany). Approximately 200 JPEG image files were obtained for each cross-sectional level, and the entire cross-section was synthesized using the free software package Image Composite Editor 64-bit 2.0 (Microsoft, Redmond, WA, USA).

### Designation of sample cross-sectional image levels, regions, and sides

Adopting the center of the medullary canal as a fixed point, each cross-section was divided equally into eight regions: the anterior (A), anterior medial (AM), medial (M), posterior medial (PM), posterior (P), posterior lateral (PL), lateral (L), and anterior lateral (AL) regions. Furthermore, each of these eight regions was defined as having both a periosteal side and an endosteal side (Fig. 1, right).

### OM evaluation

**Fig. 2:**
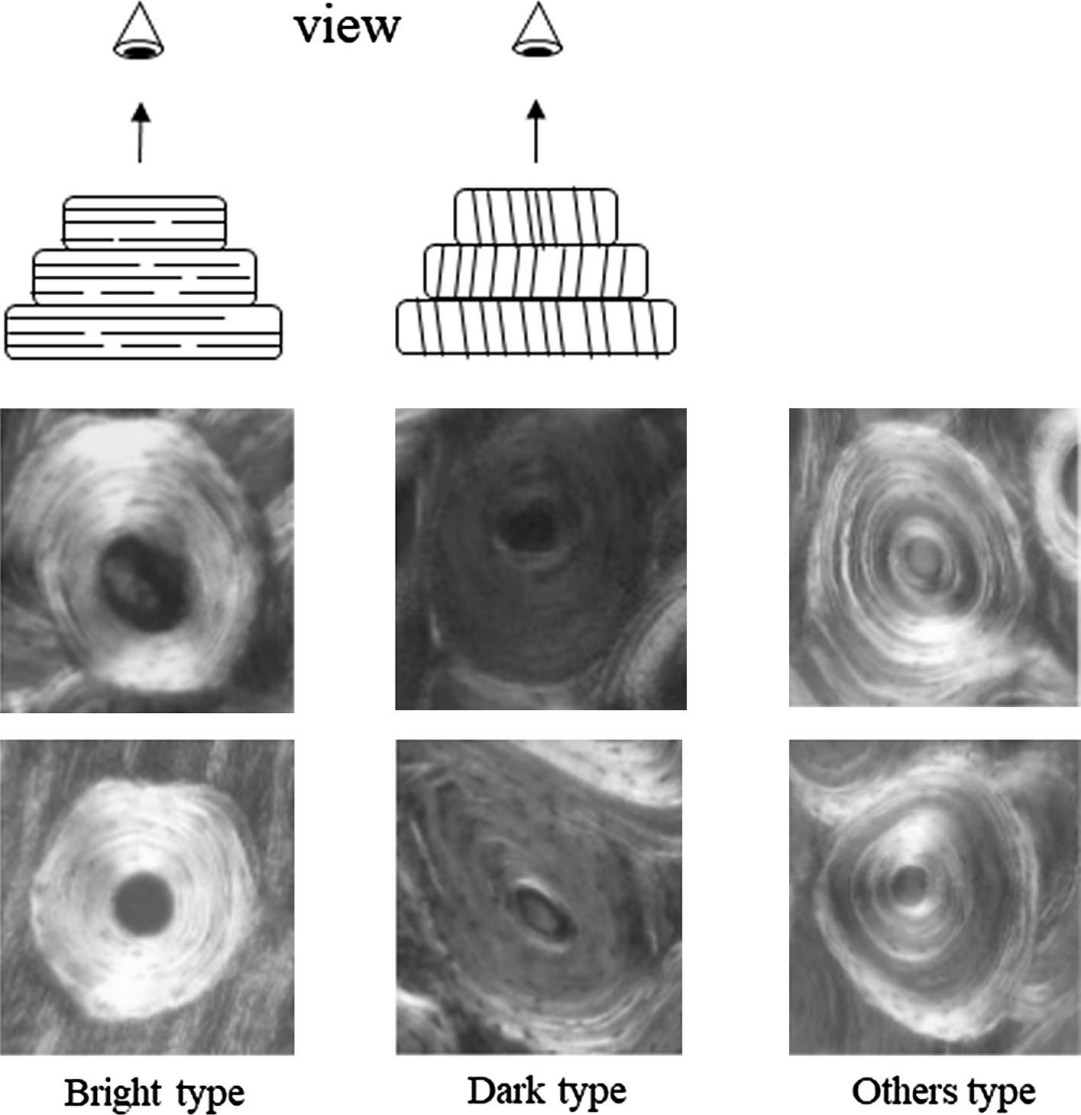
Osteon morphotype classification based on circularly polarized light microscopy imaging. The orientations of fiber bundles in successive lamellae are shown in the diagrams at the* top* of the figure (adapted from Ascenzi and Bonucci 1968). The morphotypes included a bright type (BT), a dark type (DT), and an others type (OT, characterized as types other than BT and DT). BT and DT osteons were distributed throughout the lamellar structure

**Fig. 3:**
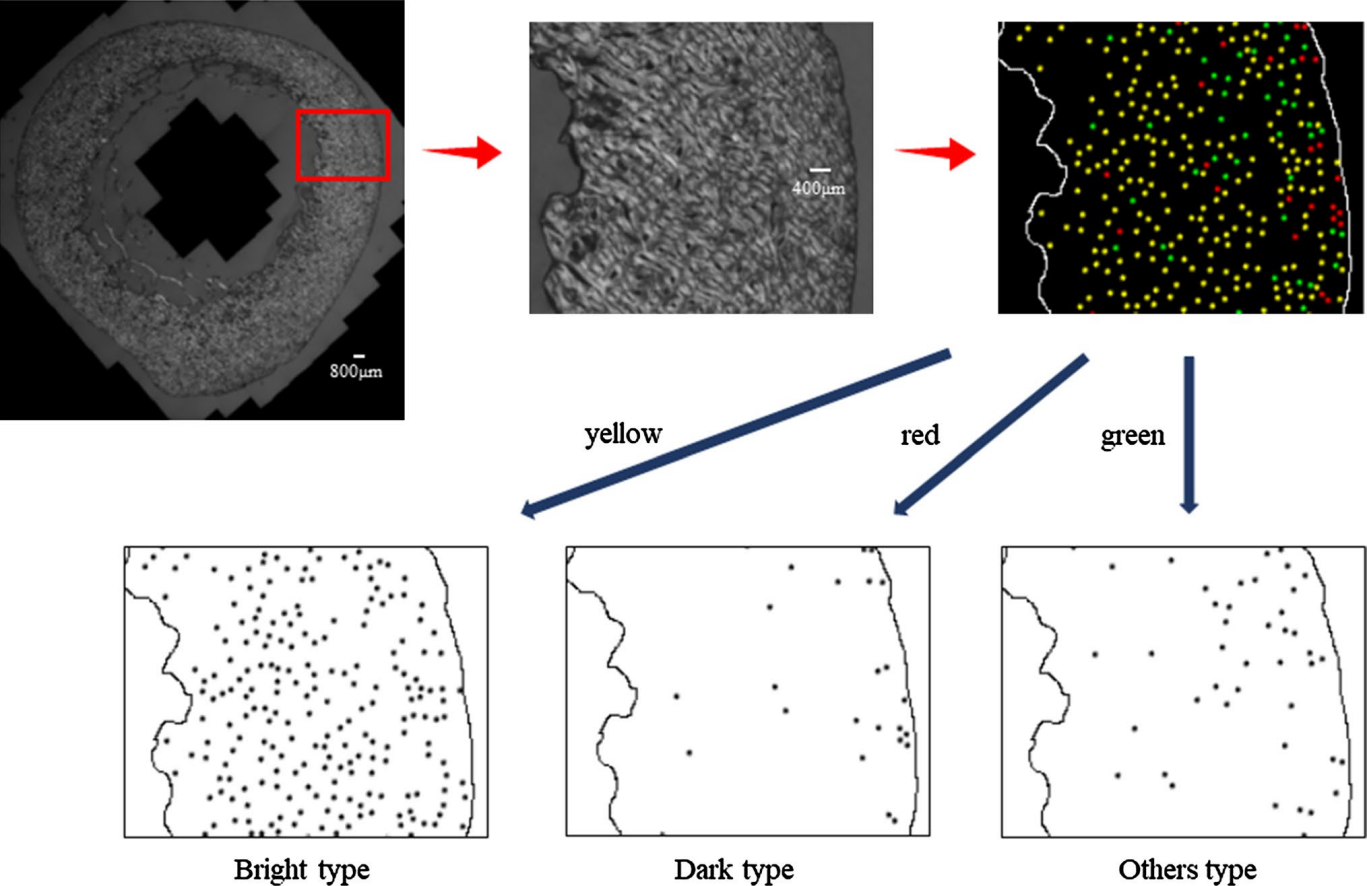
Osteon morphotype mapping on a circularly polarized light microscope image.* Yellow* bright type,* red* dark type,* green* others type

All secondary osteons present in the cross-sectional images for each level were confirmed visually on the computer screen. The osteon morphotype (OM) was determined according to the method proposed by Ascenzi and Bonucci (1968) and Bromage et al. (2003). The morphotypes were a bright type (BT), a dark type (DT), and an others type (OT), with the latter including a type with alternating light and dark layers as well as uncategorizable images (Fig. 2). Author M individually evaluated the morphotype of each osteon twice. If the results of the first and second evaluations were different, a further evaluation was performed. The secondary osteons were color coded in each image according to their morphotypes using graphical editing software (Photoshop Elements 13^®^, Adobe Systems, San Jose, CA, USA); see Fig. 3. The distribution of secondary osteons (the secondary units of bone tissue) by morphotype was examined.

1. Osteon population density

The osteon population density (OPD) is the number of osteons per unit area. We compared the OPD values in the eight regions at each cross-sectional level of each femur. The OPDs at the periosteal and endosteal sides of each specimen were also compared at each level and for each region. The change in OPD from the proximal section of the diaphysis to the distal portion was explored statistically using regression analysis.

2. OM analysis

The reproducibility of the OM discrimination results based on a comparison of multiple evaluations by the same examiner and on a comparison of the evaluations performed by two examiners was evaluated by calculating the kappa coefficient. Three sections of the acquired image was chosen at random for study by the examiners, and the two examiners independently judged the OMs of 100 secondary osteons in the same section.

The total number of osteons observed in each of the eight cross-sectional regions and at each cross-sectional level in both femurs was tabulated for each of the three OM types. The three morphotypes were compared in terms of actual numbers present, and the relative morphotype abundances were compared among cross-sectional levels.　Whether the population of BT, DT, or OT osteons exhibited an increasing or decreasing trend from the proximal level of the diaphysis to the distal level in each cross-sectional region was also determined statistically using the Cochran–Armitage trend test. Further, to clarify whether DT or BT osteons were more prevalent in each of the eight cross-sectional regions, the DT/BT ratio was calculated for each region, and differences between the regions were tested for significance using Tukey’s multiple comparisons method. DT/BT ratios on the periosteal and endosteal sides were also analyzed using the paired* t* test.

### Statistical analysis

Comparisons of the average OPD values at different levels and in different regions of the two specimens were performed using Student’s *t* test. In addition, as mentioned above, we used paired *t* tests, regression analysis, the Cochran–Armitage trend test, and Tukey’s method to statistically explore the data.

### Ethical approval

This study was approved by the ethics committee of the Nagasaki University Graduate School of Biomedical Sciences (approval number: 13102353). The rights and welfare of the cadavers in this study were protected in accordance with the ethical guidelines outlined in the Declaration of Helsinki.

## Results

**Fig. 4:**
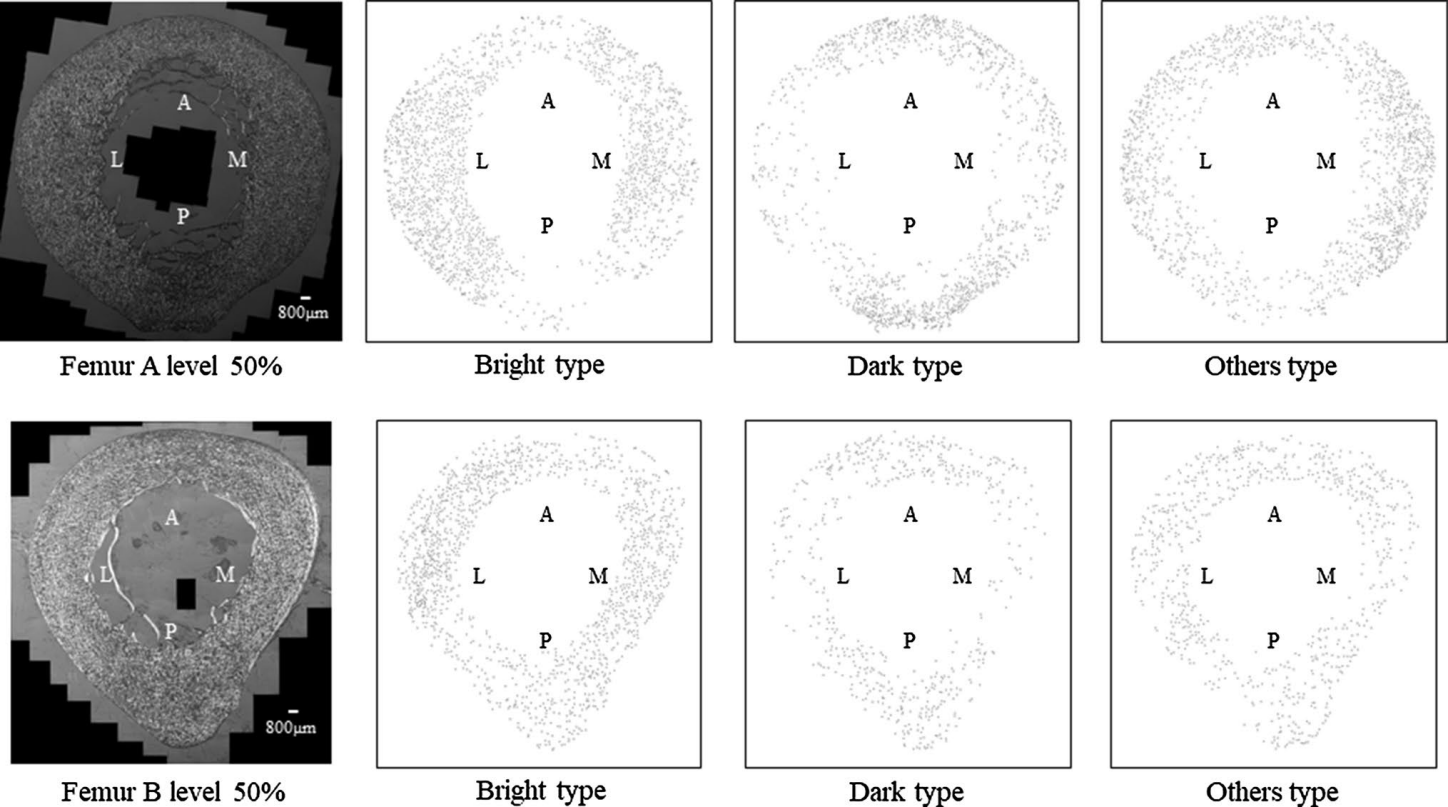
Circularly polarized light microscopic image and mapped version of that image displaying the distributions of the three osteon morphotypes at the 50% cross-sectional level. *A* anterior region, *M* medial region, *P* posterior region, *L* lateral region. It is apparent from the figure that each morphotype may tend towards a particular locus

Figure 4 displays the results of the analysis of the 50% cross-sectional levels of femurs A and B as a representative example. The results indicated that each morphotype may tend towards a particular locus.

### Osteon population density

1. Comparison of the two femurs at each level and region

**Fig. 5a–b:**
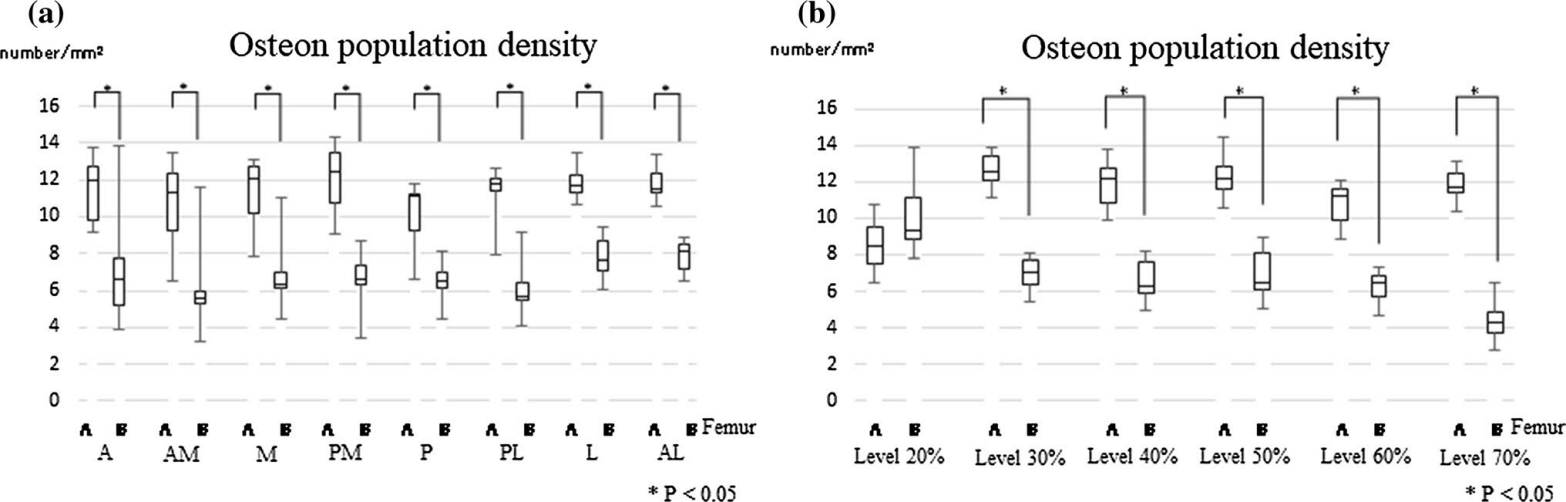
Comparison of osteon population density between femur A and femur B (**a** by region; **b** by level). *A* anterior region, *AM* anterior medial region, *M* medial region, *PM* posterior medial region, *P* posterior region, *PL* posterior lateral region, *L* lateral region, *AL* anterior lateral region. **a** Significant differences in osteon population density (OPD) were observed between femur A and femur B in all regions. **b** Significant differences between the femurs were observed at the 30% cross-sectional level and at levels more distal than the 30% level

The OPD at each level and in each region was compared between femur A and femur B. In all regions, the OPD in femur A was higher than that in femur B (Fig. 5a). At each level except for the 20% level, femur A exhibited a statistically significantly higher OPD than femur B (Fig. 5b).

2. Comparison of the OPDs on the periosteal and endosteal sides for individual specimens

In femur A, the OPD on the periosteal side was higher than that on the endosteal side in all eight regions and for all cross-sectional levels. In contrast, for femur B, there was no significant difference in OPB between the periosteal and endosteal sides for any region other than the posterior region, and there was also no significant difference in OPB between the periosteal and endosteal sides at any level aside from the 30% cross-sectional level.

3. Overall increasing or decreasing trends in femoral diaphyseal OPD by region (Table 1)

**Table 1:** Increasing or decreasing trends in femoral diaphyseal OPD (regression analysis), *p* < 0.05

Regions	Femur A	Femur B
A		↓
AM		↓
M	↑	↓
PM		↓
P		
PL		↓
L		
AL		↓

An arrow pointing upward indicates that the regression coefficient is positive from the proximal region to the distal region. An arrow pointing downward indicates that the regression coefficient is negative from the proximal region to the distal region

*OPD* osteon population density, *A* anterior region, *AM* anterior medial region, *M* medial region, *PM* posterior medial region, *P*, posterior region, *PL* posterior lateral region, *L* lateral region, *AL* anterior lateral region

A regression analysis to test for the presence of continual increases or decreases in the overall OPD of the femoral diaphysis showed that, for femur A, the bone density exhibited an increasing trend in the distal direction for the medial region only (*p* < 0.05); the other regions did not exhibit any clear trend. Meanwhile, femur B exhibited a decreasing trend in OPD in the distal direction (*p* < 0.05) for all regions except the posterior and lateral regions.

### Osteon morphotype analysis

1. Verification of the reproducibility of OM discrimination

The morphotypes of the osteons were evaluated by two examiners. The average kappa coefficient between the examiners was 0.917, and the mean interexaminer coefficient was 0.864; both of these values indicate excellent discrimination reproducibility.

2. Analysis of morphotype prevalence by level

**Fig. 6a–b:**
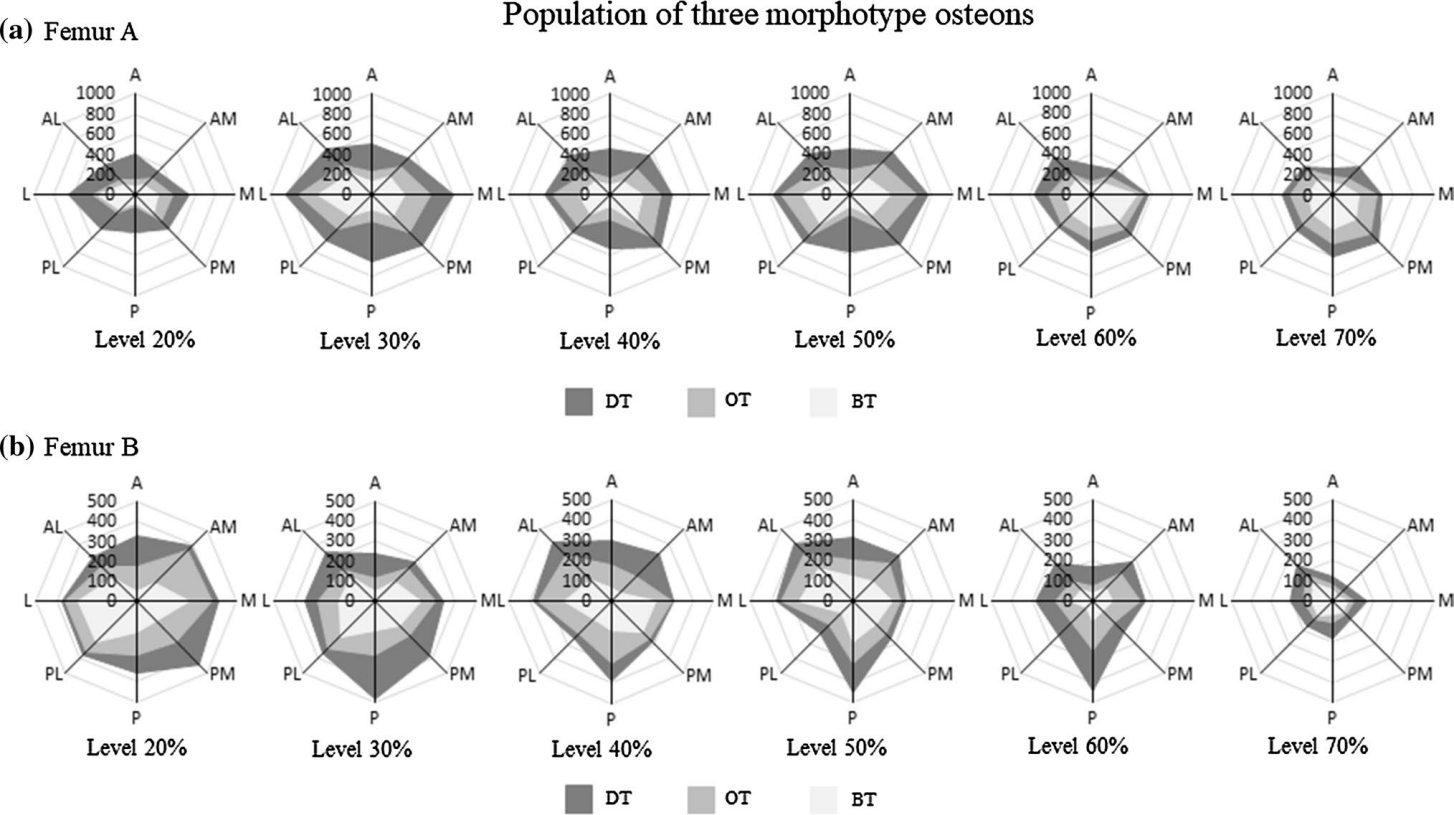
The actual number of each osteon morphotype in each region of each cross-sectional level in each femur specimen (**a** femur A; **b** femur B). The plots for femur A have an upper limit of 1000 osteons, whereas the plots for femur B have an upper limit of 500 osteons. *DT* dark type, *OT* others type, *BT* bright type

The actual number of each OM in each of the eight regions at each cross-sectional image level in each femur is displayed in the radar diagrams of Fig. 6. The plots for femur A have an upper limit of 1000 osteons and those for femur B have an upper limit of 500 osteons. For both femur A and femur B, the proportion of DT osteons was highest in the A and P regions at the 20, 30, 40, and 50% levels (more proximal than the center of the diaphysis). The BT osteons tended to be more prevalent in the M and L regions of the femur.

**Fig. 7a–b:**
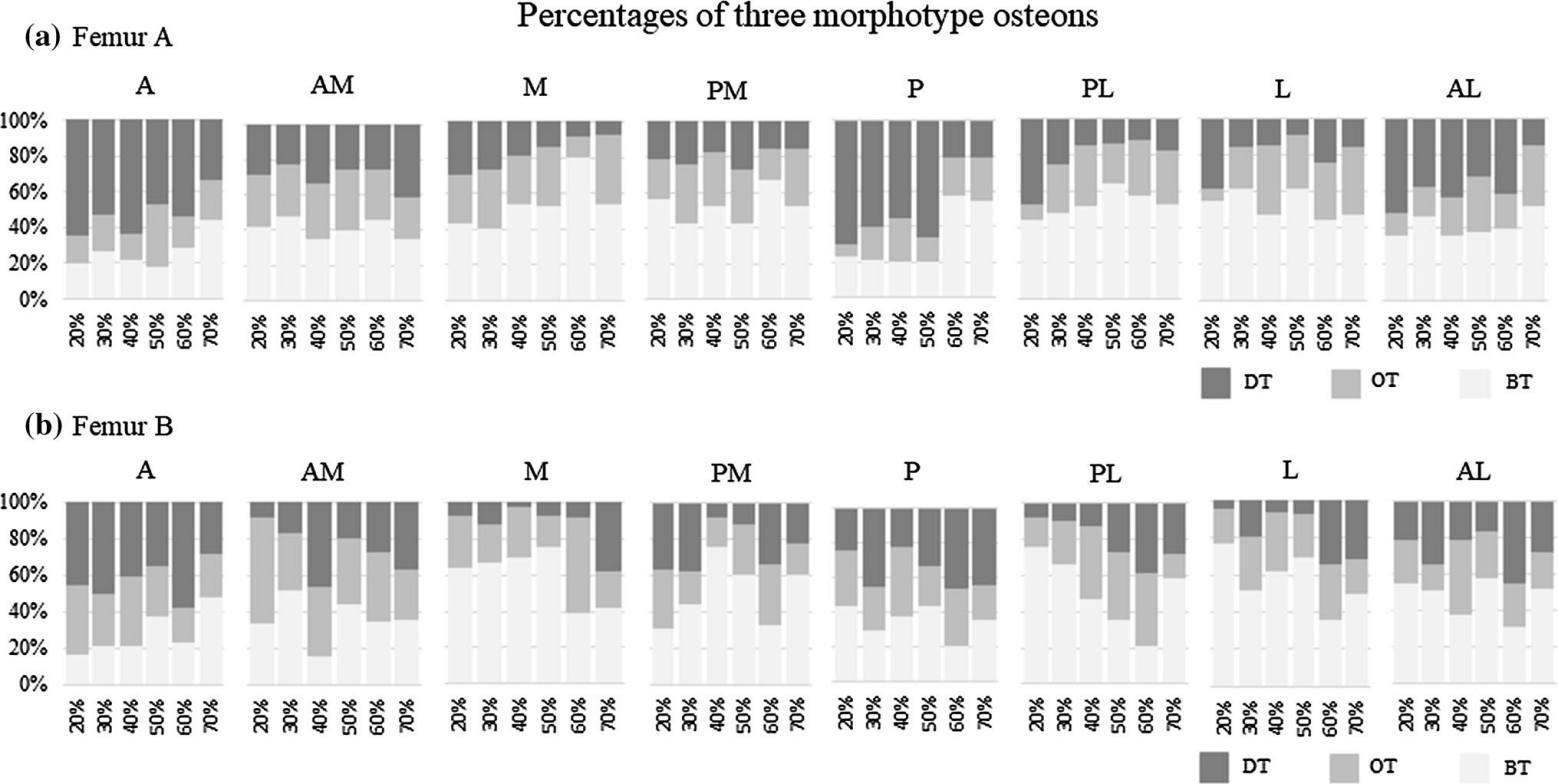
Osteon morphotype ratio by region and level (**a** femur A, **b** femur B). *A* anterior region, *AM* anterior medial region, *M* medial region, *PM* posterior medial region, *P* posterior region, *PL* posterior lateral region, *L* lateral region, *AL* anterior lateral region. *DT* dark type, *OT* others type, *BT* bright type. The variation in the osteon morphotype (OM) ratio with cross-sectional level was observed to depend on the region considered

The OM ratio for each region of each cross-sectional level in each femur is displayed in the stacked bar graphs of Fig. 7. To explore variations in the OM ratio across cross-sectional levels in each femur, the proximal to distal regions were evaluated using the Cochran–Armitage trend test. The relative abundance of DT osteons in femur A exhibited a decreasing trend in the distal direction in the A, M, P, PL, and AL regions. In contrast, with the exception of the L region, the relative abundance of BT osteons increased in the distal direction in a wide range of regions. Thus, in femur A, the relative abundance of DT osteons was highest in proximal regions, while the relative abundance of BT osteons tended to increase in distal regions, particularly in the A to the M regions (Fig. 7a).

For femur B, although the relative abundance of DT osteons exhibited an increasing trend from the proximal region to the distal region except in the A, PM, and AL cross-sectional regions, the trend in BT osteon prevalence varied from region to region (Fig. 7b). Thus, the patterns of morphotype prevalence in the proximal and distal regions of the diaphyseal segments of femurs A and B exhibited almost opposite tendencies (Table 2).

**Table 2:** Increasing or decreasing trends in the prevalences of DT, BT, and OT osteons in the femoral diaphysis (Cochran–Armitage trend test), *p* < 0.05

Region	Femur A			Femur B		
	DT	BT	OT	DT	BT	OT
A	↓	↑	↑		↑	↓
AM				↑		↓
M	↓	↑		↑	↓	
PM		↑			↑	
P	↓	↑	↑	↑	↓	
PL	↓	↑	↑	↑	↓	↑
L		↓		↑	↓	
AL	↓	↑	↑			

An arrow pointing upward indicates that the regression coefficient is positive from the proximal region to the distal region. An arrow pointing downward indicates that the regression coefficient is negative from the proximal region to the distal region

*A* anterior region, *AM* anterior medial region, *M* medial region, *PM* posterior medial region, *P* posterior region, *PL* posterior lateral region, *L* lateral region, *AL* anterior lateral region, *DT* dark type, *BT* bright type, *OT* others type

3. The DT/BT ratio on the periosteal and endosteal sides

**Fig. 8a–d:**
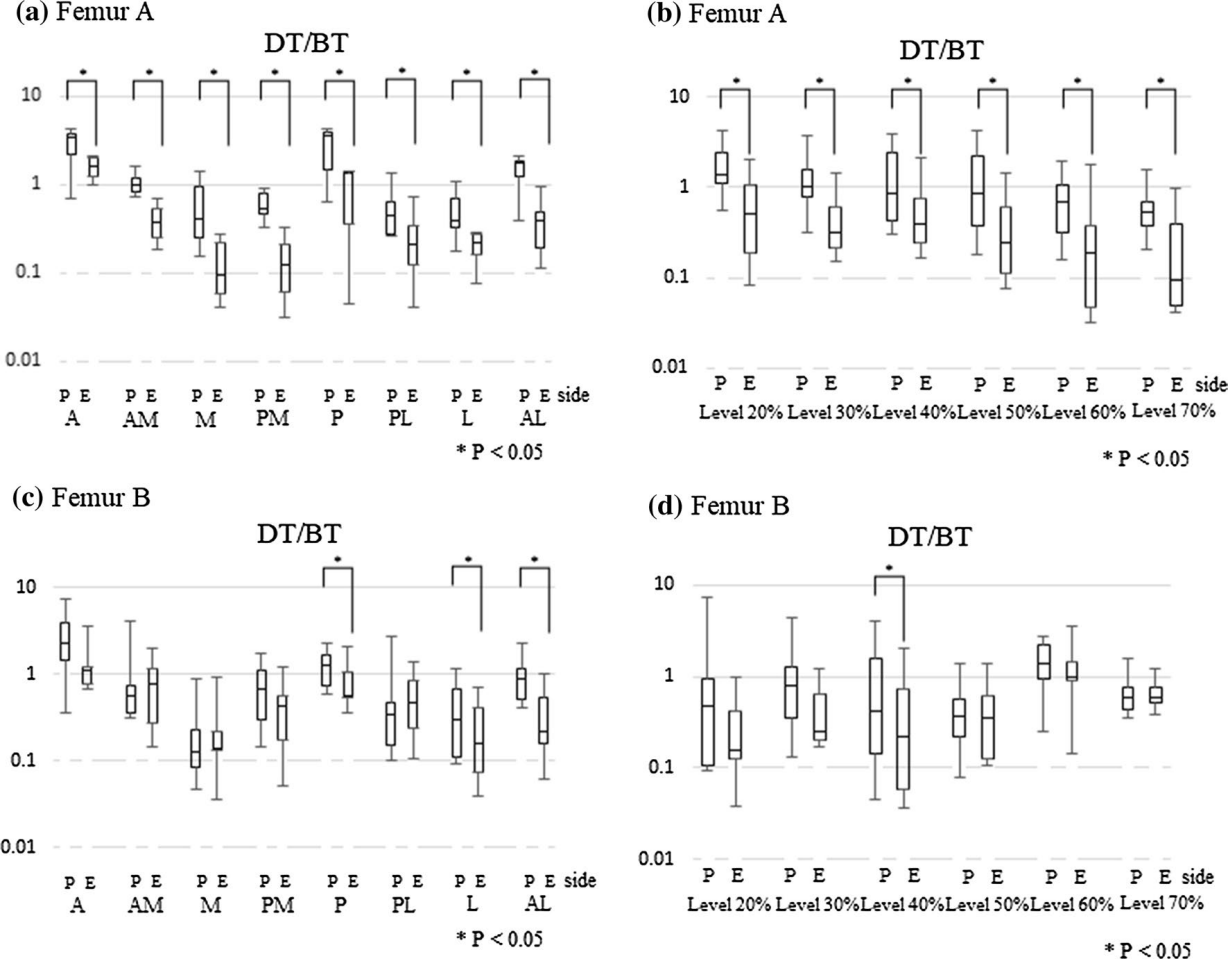
The DT/BT values for the periosteal and endosteal sides of each femur (**a**, **c** by region; **b**, **d** by level). *A* anterior region, *AM* anterior medial region, *M* medial region, *PM* posterior medial region, *P* posterior region, *PL*, posterior lateral region, *L* lateral region, *AL* anterior lateral region. Sides: *P* periosteal side, *E* endosteal side. *DT* dark type, *BT* bright type. In both femurs, the DT/BT ratio was high in regions A and P (**a**, **c**). In femur A, the DT/BT ratio on the periosteal side was higher than on the endosteal side at all levels and in all regions (**a**, **b**)

The DT/BT ratio was quantified on the periosteal and endosteal sides of femurs A and B at each level and in each region (Fig. 8). Because there was significant distortion of the DT/BT ratio distribution, the plots in Fig. 8 are shown on a logarithmic scale. For femur A, in particular, the DT/BT ratio on the periosteal side was higher than that on the endosteal side (Fig. 8a, b). Meanwhile, in femur B, the DT/BT ratio on the periosteal side was highest in the P, L, and AL regions (Fig. 8c) and at the 40% level (Fig. 8d). Both femurs showed a tendency to have higher DT/BT ratios in the A and P regions than in the other cross-sectional regions of the femoral diaphysis (Fig. 8a, c). Using Tukey’s multiple comparison test, the DT/BT ratio was found to be higher in the A and P regions (*p* < 0.05) of both femurs.

## Discussion

### Osteon population density

Femur A (estimated to be 53 ± 3 years old) exhibited a higher OPD than femur B (estimated to be 33 ± 3 years old) in all regions and at several cross-sectional levels. Although femur B exhibited no significant difference in OPD between the periosteal and the endosteal sides, the OPD tended to increase in the proximal direction in several regions at various cross-sectional levels. These findings indicate that in femur B, the prevalence of secondary osteons depended on the cross-sectional level of the femoral diaphysis. It can be inferred that since cortical bone turnover is abundant in high-OPD regions (Miszkiewicz 2016), the degree of adaptation to mechanical load forces may differ between the distal and proximal regions of the femur.

In contrast, almost all of the cross-sectional levels in femur A exhibited no increasing or decreasing trend in OPD, and the OPD was found to be evenly distributed from the proximal region to the distal region. However, a comparison of the OPDs on the periosteal and endosteal sides revealed that the OPD on the periosteal side was higher in all regions. As no significant difference in OPD was observed between the periosteal and endosteal sides in femur B, we can conclude that osteons which form in conjunction with aging are likely to be more prevalent on the periosteal side. Gocha and Agnew (2016) reported that the OPD on the periosteal side is higher in individuals aged 50 years or older, and the results of our study are consistent with their observation.

### Osteon morphotype analysis

Each osteon morphotype has its own mechanical characteristics. Longitudinal-type (dark-type) osteons exhibit high resistance to tensile forces, while transversal-type (bright-type) osteons have been shown in numerous studies to exhibit resistance to compression forces (Ascenzi and Bounucci 1967, 1968, 1972). Each osteon examined in this study was observed directly under a microscope, and the morphology of each individual osteon was determined. The results of this examination were shown to be reproducible, and we believe that these results reflect the actual situation.

Aspects that the femur specimens from donors of different ages shared included the existence of DT osteon concentrations at muscle attachment sites, which is believed to be due to the presence of the anterior region and the posterior region (i.e., the proportion of osteons that were adapted to pull forces was higher than in other areas). Based on these observations, we can infer that there is a tendency for osteons that are most capable of resisting pull forces to proliferate in these areas, particularly in the linea aspera region. In both femurs, we observed a tendency for BT osteons to be more prevalent in the medial and lateral regions of each cross-section. This suggests that the medial and lateral regions tend to be exposed to the strongest compression forces. Duda et al. (1997, 1998) conducted an evaluation of the biomechanical capacity of the human femur and found that different forces were exerted on different regions of the bone. They also reported that the tensile forces on the femur are stronger than the compressive forces generated at the anterior and lateral regions. Polgár et al. (2003) reported that tensile forces are exerted on the femoral lateral region while compressive forces are stronger in the medial region. Though the results obtained in our study did not completely support those findings, it was assumed that the main external force in both the medial and lateral regions was compressive load.

Furthermore, with regard to differences between the two femurs by level, femur A had DT osteons occupying large regions at the proximal levels and BT osteons occupying large regions at the distal levels. Such trends were apparent throughout the entire specimen, particularly from the anterior to the medial regions. In contrast, the trends in the BT and DT osteon prevalences for femur B were essentially the reverse of those for femur A: osteons classified as BT were particularly abundant at the proximal level whereas osteons classified as DT were especially prevalent at the distal level. It is known that the muscles attached to the femur can affect the mechanical load exerted on the femur (Duda et al. 1997; Rybicki et al. 1972), and the two femur specimens exhibited different OM prevalence trends. This suggests the possibility that different external forces were applied to the femoral diaphysis. There have been reports that transverse collagen fiber presence increases with age (Smith 1960; Vincentelli and Evans 1971), but, because there is a possibility that these studies did not include a comprehensive assessment of the entire femur, further examination is necessary.

### Differences in cortical bone quality between the femurs

Although this study focused on only two femur specimens, detailed examinations indicated that there were large interindividual differences in OPD and OM prevalence. Assuming that each donor was a representative example of their respective age cohort, several inferences can be drawn from our results. Concerning the OPD, the osteon density was higher in the younger femur (B), as the region examined was more proximal. In contrast, the shift in OPD across levels seen in femur B was not apparent in the older femur (A). In essence, this observation suggests that secondary osteons which increase with age may tend to form in the distal regions of the diaphysis. Considering that we observed a tendency for the relative abundance of BT osteons to increase in the distal diaphysis in femur A, we drew the following inferences: the process of additional osteon formation that occurs in conjunction with aging leads to the proliferation of osteon types that are resistant to compression forces primarily in the periosteal region of the distal femoral diaphysis; cortical bone quality can influence the fragility of the diaphyses of long bones; and pathological conditions such as fragility fractures tend to occur in regions of the diaphysis where a breakdown in this morphological adaptive process or osteon level adaptation occurs. There is also the possibility that the differences observed between these two femur specimens were the result of interindividual differences (e.g., in age), and future studies are necessary to validate our results by increasing the number of specimens examined.

### Study limitations

The first limitation of this study was its small sample size, as femur specimens were taken from only two individuals. Second, there was no assessment of any potential impact of specimen background characteristics, such as donor, height, weight, or medical history. To fully clarify how age-related changes affect the entire femoral diaphysis, it will be necessary to increase the number of specimens examined and comprehensively analyze the femoral cortex.

## Conclusion

The orientation and force of the mechanical load on the diaphyseal region of cortical bone tissue are believed to change dynamically throughout a day of activity. Although the endoskeleton of a vertebrate can change its morphology, as described by Wolff’s law, it is presumed that, even at the microstructural level, the osteon distribution pattern also changes in response to such adjustments. Osteons may also counteract mechanical burdens by adapting their intrinsic collagen sequence. Although this study compared specimens taken from only two human femurs obtained from donors of different ages, our results demonstrate that there is diversity among individuals with respect to the OPD distribution and the OM ratio. It is expected that, by analyzing the morphological aging of cortical bone and the collagen sequences of the many osteons that constitute the cortical bone tissue at the same time, we will be able to improve our understanding of osteon microstructure.
